# Secretory expression of biologically active human Herpes virus interleukin-10 analogues in *Escherichia coli* via a modified Sec-dependent transporter construct

**DOI:** 10.1186/1472-6750-13-82

**Published:** 2013-10-05

**Authors:** Sarah Förster, Manuela Brandt, Dorothea S Mottok, Anke Zschüttig, Kurt Zimmermann, Frederick R Blattner, Florian Gunzer, Christoph Pöhlmann

**Affiliations:** 1 Institute of Medical Microbiology and Hygiene, TU Dresden, Fiedlerstrasse 42, 01307 Dresden, Germany; 2 QIAGEN Hamburg GmbH, Königstrasse 4a, 22767 Hamburg, Germany; 3 SymbioPharm GmbH, Auf den Lüppen 8, 35745 Herborn-Hörbach, Germany; 4 Scarab Genomics LLC, 1202 Ann St., 53713 Madison, Wisconsin, USA; 5 Department of Genetics, University of Wisconsin, 425G Henry Mall, 53706-1580 Madison, Wisconsin, USA; 6 Department of Laboratory Medicine, Robert-Bosch Hospital, Auerbachstrasse 110, 70376 Stuttgart, Germany

**Keywords:** *Escherichia coli*, Interleukin-10, Outer membrane protein F, Inflammatory bowel disease, Bacterial transport system

## Abstract

**Background:**

Interleukin-10 homologues encoded by Herpes viruses such as Epstein-Barr virus (EBV) and human cytomegalovirus (HCMV) hold interesting structural and biological characteristics compared to human interleukin-10 (hIL-10) that render these proteins promising candidates for therapeutic application in inflammatory bowel disease (IBD). Intestinal delivery of cytokines using bacterial carriers as chassis represents a novel approach for treatment of IBD patients. For proof of concept, a Sec-dependent transporter construct was designed for secretory expression of recombinant viral IL-10 proteins in the periplasm of *Escherichia coli* laboratory strain BL21 (DE3), which might serve as part of a prospective lysis based delivery and containment system.

**Results:**

The signal peptide of *E. coli* outer membrane protein F fused to the mature form of the viral IL-10 proteins enabled successful transport into the periplasm, a compartment which seems crucial for proper assembly of the dimeric configuration of the cytokines. Cytokine concentrations in different bacterial compartments were determined by ELISA and achieved yields of 67.8 ng/ml ± 24.9 ng/ml for HCMV IL-10 and 1.5 μg/ml ± 841.4 ng/ml for EBV IL-10 in the periplasm. Immunoblot analysis was used to confirm the correct size of the *E. coli*-derived recombinant cytokines. Phosphorylation of signal transducer and activator of transcription 3 (STAT3) as part of the signal transduction cascade after IL-10 receptor interaction, as well as suppression of tumor necrosis factor α (TNF-α) release of lipopolysaccharide-stimulated mouse macrophages were used as read-out assays for proving *in vitro* biological activity of the *E. coli* derived, recombinant viral IL-10 counterparts.

**Conclusions:**

In this study, proof of principle is provided that *E. coli* cells are a suitable chassis for secretory expression of viral IL-10 cytokines encoded by codon-optimized synthetic genes fused to the *E. coli omp*F signal sequence. *In vitro* biological activity evidenced by activation of transcription factor STAT3 and suppression of TNF-α in mammalian cell lines was shown to be strictly dependent on export of viral IL-10 proteins into the periplasmic compartment. *E. coli* might serve as carrier system for *in situ* delivery of therapeutic molecules in the gut, thus representing a further step in the development of novel approaches for treatment of IBD.

## Background

Inflammatory bowel disease (IBD), such as Colitis ulcerosa and Crohn’s disease, present with symptoms like diarrhoea, abdominal pain and weight loss which often means a long ordeal for the patients as well as a great effort for the health care system. Though wide parts of the underlying pathogenesis are still unknown, main components have been described and open up the opportunity for novel therapeutic approaches. Profound downregulation of the auto-inflammatory process has recently been attempted with antibodies against tumor necrosis factor α (TNF-α) [1]. A totally new conception is the *in situ* delivery of the anti-inflammatory cytokine interleukin-10 (IL-10) via bacterial carrier systems. Steidler et al. showed decreased inflammation in chemically induced colitis of mice treated with a *Lactococcus lactis* strain secreting murine IL-10 [2].

Since human IL-10 (hIL-10) possesses not only anti-inflammatory properties like down-regulation of pro-inflammatory cytokines, inhibition of antigen presentation on dendritic cells or suppression of major histocompatibility complex expression, but also displays pro-inflammatory activity such as stimulation of B-cell maturation and proliferation of natural killer cells [3], IL-10 homologues encoded by members of the Herpes virus family move into the focus of interest.

Human cytomegalo- (HCMV) and Epstein-Barr virus (EBV) perfected their strategies to avoid eradication by the immune system during co-evolution with the host [4]. The EBV and HCMV IL-10 counterparts encoded by the BCRF1 and UL111A gene region, respectively, enable Herpes viruses among other mechanisms to escape the host’s immune system and to establish a latent, lifelong infection. Viral IL-10 homologues share many biological activities of hIL-10 but, due to selective pressure during virus evolution, also display unique traits such as increased molecule stability and lack of immunostimulatory functions [5-7]. These characteristics suggest the viral counterparts to be even more effective than hIL-10 as immunosuppressants. Thus, recombinant viral IL-10 (vIL-10) proteins emerge as promising candidates for therapeutic applications. So far, only EBV IL-10 has been successfully expressed in both, prokaryotic and eukaryotic expression systems, which, however, required further steps to yield a functional protein [8,9].

We aim at using *Escherichia coli* as chassis for intestinal delivery of recombinant vIL-10 proteins in IBD patients. In a recent study, we have demonstrated that the bacterial periplasm is a suitable milieu for expression of biologically active recombinant IL-10 [10]. An inducible cell lysis device may then confer both, biological containment and release of IL-10 into the culture medium [11]. Thus, as proof of concept, a Sec-dependent vIL-10 transporter was constructed in laboratory *E. coli* strain BL21 (DE3) which allows secretory expression of codon optimized viral IL-10 genes in the *E. coli* periplasm. Translocation of recombinant viral proteins into *E. coli* periplasm was achieved by fusing the signal peptide of the *E. coli* outer membrane protein F (OmpF) to the mature form of the vIL-10 proteins. The biological activity of the recombinant viral proteins was proved by two independent cell-based *in vitro* assays. To our knowledge, we describe here for the first time the successful secretory expression of biologically active viral IL-10 homologues in a prokaryotic chassis without further purification steps.

## Results and discussion

### Design and cloning of the artificial vIL-10 transporters

An *E. coli* codon optimized nucleotide sequence was generated from the viral IL-10 gene sequences (HCMV IL-10: 477 bp, GenBank accession number 1LQS_M; EBV-IL10: 441 bp, GenBank accession number YP_401634). The original signal sequences of the viral IL-10 genes were replaced by the first 66 nucleotides of the *ompF* gene of *E. coli* K12-MG1655 (GenBank accession number NC_000913.2) coding for the 22 aa long OmpF signal peptide. Using SignalP 3.0 software (http://www.cbs.dtu.dk/services/SignalP/, Technical University of Denmark), a high cleavage probability (score of 0.998) was predicted between aa position 22 and 23 (ANA^22^ – S^23^E for HCMV IL-10 and ANA^22^ – Q^23^C for EBV IL-10) delivering the mature form of the vIL-10 proteins with authentic N-terminus.

**Figure 1:**
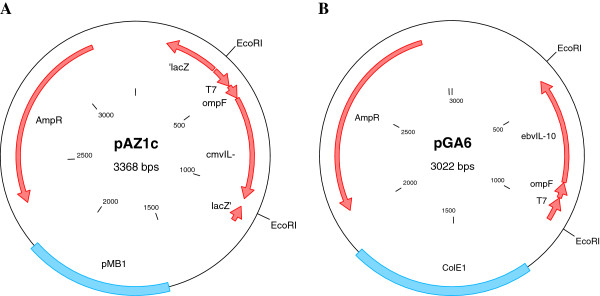
**Plasmid maps of HCMV IL-10 (pAZ1c; A) and EBV (pGA6; B) expression vectors are depicted.** The artificial transporter consists of the *E. coli ompF* signal sequence fused in frame to *E. coli* codon optimized mature viral IL-10 genes under control of the T7 promoter. For subcloning, the constructs are flanked by *Eco*RI restriction sites. Plasmid pGA6 contains a ColE1, pAZ1c a pUC19-derived pMB1 origin of replication.

The *E. coli* ompF gene was fused in-frame to the 5’ end of the *E. coli* codon optimized gene encoding HCMV IL-10 and EBV IL-10, respectively. The expression of both synthetic gene constructs was regulated by a T7 promoter. The complete sequence of the artifical vIL-10 transporters including the T7 promoter sequence was synthesized by GeneArt (Regensburg, Germany) and cloned into a pUC-derived plasmid with ColE1 origin resulting in the plasmids pGA4 encoding the HCMV IL-10 transporter and pGA6 carrying the EBV IL-10 construct, respectively. The vIL-10 transporter constructs were subsequently subcloned via an *Eco*RI restriction site into pUC19 vector with pMB1 origin. The new expression vectors, pAZ1c, a derivative of pGA4, and pAZ1e, a derivative of pGA6, were isolated and sequenced. *E. coli* BL21 (DE3) cells were transformed with pGA4 and its corresponding plasmid pAZ1c as well as with pGA6 and the according plasmid pAZ1e. Plasmid maps of the vectors pAZ1c and pGA6 used in the STAT3 and TNF-α read-out assays are shown in Figure 1.

### Quantification of recombinant vIL-10 expression

The concentration of the viral IL-10 analogues expressed and secreted *in vitro* by *E. coli* strain BL21 (DE3) containing either plasmid pGA4, pGA6, pAZ1c or pAZ1e were determined by sandwich ELISA. After overnight cultivation of the transformed strains, culture supernatant was obtained by centrifugation while the periplasmic compartment was separated from the cytoplasm by using the osmotic shock procedure as described by Neu and Heppel [12]. Due to high homology on protein level between EBV IL-10 and hIL-10 [13], detection of recombinant EBV IL-10 was possible using a commercial hIL-10 ELISA from R&D Systems (Minneapolis, MN, USA). For quantification of recombinant HCMV IL-10 in different bacterial cell compartments, an in-house sandwich ELISA according to Chang’s description [14] was established (see Methods).

The mean concentrations of recombinant HCMV IL-10 obtained from an overnight culture of pAZ1c transformed *E. coli* BL21 (DE3) were 18.4 ng/ml ± 12.4 ng/ml for the culture supernatant and 67.8 ng/ml ± 24.9 ng/ml for the periplasm. Interestingly, in the cytoplasm 18.7 μg/ml ± 12.8 μg/ml of recombinant HCMV IL-10 could be detected. The mean concentrations of recombinant HCMV IL-10 found in different cell compartments of pGA4 transformed *E. coli* BL21 (DE3) showed three times lower levels of recombinant protein (data not shown). Plasmid pGA4 contains a ColE1 origin while pAZ1c, which is based on expression vector pUC19, harbors a pMB1 origin, thus resulting in a different copy number per cell. This may be one reason for the observed difference in the measured recombinant HCMV IL-10 concentrations. Since the opposite effect was observed for the EBV IL-10 expression vectors pAZ1e and pGA6, the compatibility of the vIL-10 transporter constructs with the different origins of replication present on the two vector backbones may play a role, too. Due to the higher yield of recombinant HCMV IL-10 protein from expression vector pAZ1c, all subsequent experiments were performed with this plasmid.

The concentration of recombinant EBV IL-10 of pGA6 transformed *E. coli* BL21 (DE3) were 1.2 ng/ml for culture supernatant, 1.5 μg/ml ± 841.4 ng/ml for the periplasmic fraction and 27.8 ng/ml ± 43.4 ng/ml for the cytoplasm. The concentrations of recombinant EBV IL-10 obtained from overnight cultures of pAZ1e transformed *E. coli* BL21 (DE3) were significantly lower (data not shown), so all further experiments were carried out with expression vector pGA6. The vIL-10 concentrations determined for the different bacterial compartments are summarized in Table 1.

**Table 1:** **Viral IL-10 concentrations in different cell compartments of *E. coli *BL 21 (DE3)**

**Viral IL-10 concentration (ng/ml ± SD)**	**HCMV IL-10 expression plasmid pAZ1c**	**EBV IL-10 expression plasmid pGA6**
**Supernatant**	**8.4 ± 12.4**	**1.2 ± n.d.**
**Periplasm**	**67.8 ± 24.9**	**1500 ± 841.4**
**Cytoplasm**	**18700 ± 12800**	**27.8 ± 43.4**

Recombinant vIL-10 concentrations from overnight cultures of *E. coli* BL 21 (DE3) transformed with either pAZ1c (HCMV IL-10) or pGA6 (EBV IL-10) are given for different bacterial compartments. Quantification of vIL-10 expression was accomplished by use of a commercial human IL-10 (for EBV IL-10) and an in-house sandwich ELISA (for HCMV IL-10), respectively. SD = standard deviation; n.d. = not done.

A number of signal sequences have been employed for efficient secretory production of heterologous proteins in *E. coli* via the Sec-dependent secretion pathway, including pectate lyase B (PelB), outer membrane protein A (OmpA), alkaline phosphatase (PhoA), endoxylanase, and heat-stable enterotoxin 2 (StII) [15]. The N-terminal portion of OmpF, a major outer membrane porin of *E. coli*, has also been successfully applied for secretion of foreign gene products in *E. coli*[16]. Most recently, the OmpF leader peptide was successfully used for secretory expression of biologically active hIL-10 in the periplasm of *E. coli* cells [10].

By fusing the OmpF signal sequence encompassing the first 22 aa of the OmpF protein to the mature form of the vIL-10 proteins, an artificial transporter was created which allowed translocation of both recombinant vIL-10 proteins across the cytoplasmic membrane, thus showing that the modified transporter was in general compatible with the host secretion apparatus. However, the efficiency of the Sec-dependent preprotein translocation pathway was different for the two viral IL-10 molecules. Expression of recombinant EBV IL-10 in *E. coli* strain BL21 (DE3) showed much higher protein concentrations in the periplasm compared to recombinant HCMV IL-10, for which the concentration in the cytoplasm was much higher than in other bacterial compartments. Thus, it seems that the OmpF-EBV IL-10 preprotein is more compatible with the Sec translocase machinery than the corresponding HCMV IL-10 molecule. Of note, the recombinant vIL-10 proteins also appeared in the culture medium, reaching an amount, which was not negligible. Given the high EBV IL-10 concentration gradient across the outer cell membrane, the secretion of the recombinant protein into the culture medium was not quantitative. Release of recombinant HCMV IL-10 was much higher compared to *E. coli*-derived EBV IL-10, thus providing an explanation for the lower concentration of recombinant HCMV IL-10 protein measured in the periplasm compared to recombinant EBV IL-10.

Whether secretion of vIL-10 proteins into the culture medium was selective remained unknown since the amount of representative periplasmic proteins such as β-lactamase or alkaline phosphatase was not assessed. The outer membrane of *E. coli* contains proteins such as OmpF, OmpC and others which serve as passive diffusion pores for small hydrophilic molecules [17]. However, the pores are too small for the vIL-10 molecules to be released (mol. wt. of the HCMV and EBV IL-10 monomer is 18 and 21 kDa, respectively [18,19]) since the pores’ exclusion limit is a molecular weight of approximately 700 Da [16]. Thus, the mechanism by which recombinant vIL-10 proteins were translocated across the outer membrane remained unknown. Leakage out of destroyed bacterial cells would be a reasonable explanation, but would suggest approximately equal amounts of both recombinant vIL-10 proteins in the culture supernatant.

### Immunoblot analysis of *E. coli*-derived vIL-10 proteins

**Figure 2:**
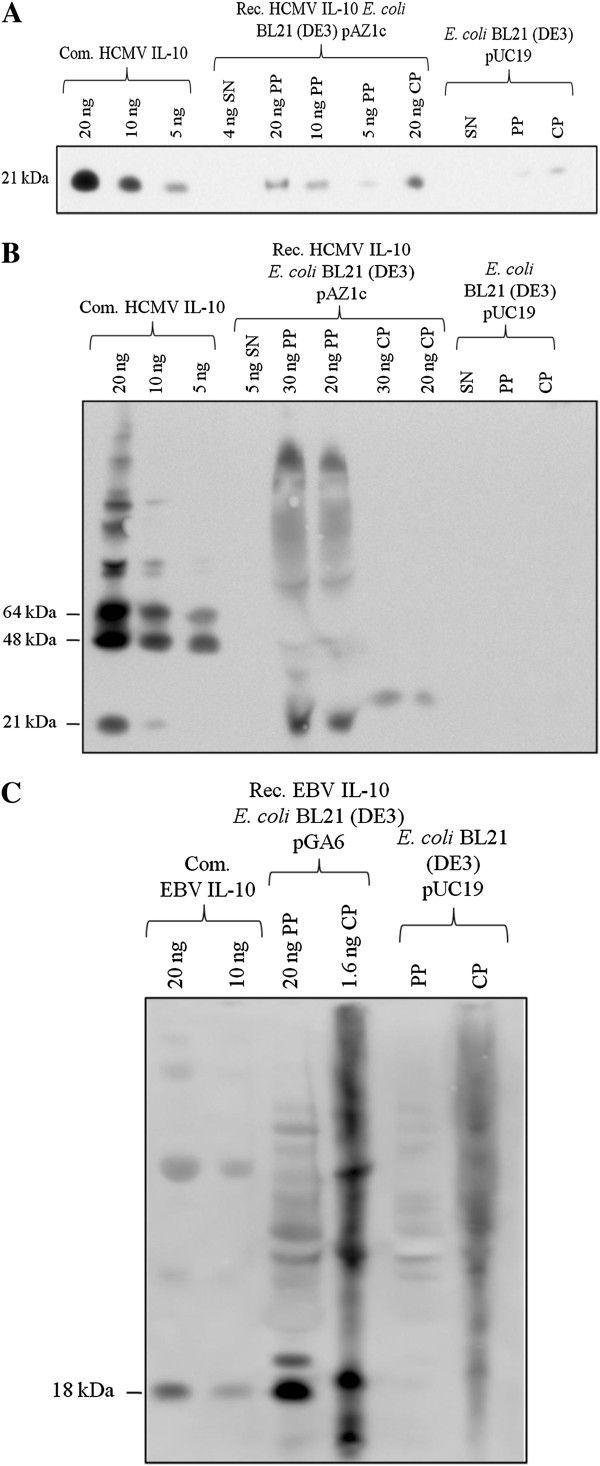
**Immunoblot analysis of viral IL-10 recombinant proteins. ***E. coli* BL21 (DE3) derived recombinant viral IL-10 proteins were analyzed by immunoblot in different concentrations and from different cell compartments under both reducing (**A** = HCMV IL-10; **C** = EBV IL-10) and non-reducing conditions (**B** = HCMV IL-10). Periplasmic and cytoplasmic fractions of pUC19 transformed *E. coli* BL21 (DE3) cells and commercial viral IL-10 proteins served as controls. One experiment representative of three is shown. SN = supernatant; PP = periplasmic fraction; CP = cytoplasmic fraction; Com. = commercial; Rec. = recombinant.

To further substantiate the results obtained from the ELISA analysis, Western immunoblot assays were carried out with culture supernatant, peri- and cytoplasmic fractions of pAZ1c and pGA6 transformed *E. coli* BL21 (DE3) strains. By using the mature forms of the vIL-10 proteins as commercial standard, the size of the *E. coli*-derived recombinant vIL-10 proteins was estimated. Bacterial fractions of pUC19 transformed *E. coli* BL21 (DE3) served as negative controls. Figure 2 shows a Western blot of the periplasmic fractions of *E. coli* BL21 (DE3) harboring either the expression vector pAZ1c or pGA6. In the periplasm, vIL-10 products corresponding to the size of the commercial proteins can be observed, indicating correct expression of the *E. coli* codon optimized vIL-10 genes. The *E. coli*-derived HCMV and EBV IL-10 proteins migrated at a molecular size of approximately 21 and 18 kDa, respectively, reflecting the size of the vIL-10 monomers (Figure 2A and 2C). In the reducing SDS-PAGE immunoblot, the HCMV IL-10 from the bacterial cytoplasmic fraction migrated at a similar molecular size compared to the commercial standard and the recombinant protein from the periplasmic fraction (Figure 2A), whereas under non-reducing conditions it ran at a slightly larger size than the commercial standard and the periplasmic equivalent (Figure 2B). The non-reducing conditions of the immunoblot might be responsible for this visible shift due to a change in the electrophoretic mobility of the full-length HCMV IL-10 protein. HCMV IL-10 protein concentrations in the culture supernatant were too low to be detectable by the immunoblot technique. Western blot analysis of the supernatant fraction containing *E. coli*-derived recombinant EBV IL-10 was not carried out.

Whether or not the OmpF signal peptide has been processed during the translocation across the inner membrane cannot be judged precisely due to the low resolution of the immunoblot gel. However, *in vitro* biological activity could be proven, thus indicating either a correct processing of the pre-protein or that the uncleaved signal peptide does not affect the IL-10 – IL-10 receptor interaction.

It has been shown that within the IL-10 subfamily of cytokines only HCMV IL-10 monomers are covalently linked via a disulfide bond [20]. Thus, immunoblot analysis under non-reducing conditions is suitable to prove whether the dimeric state of the *E. coli*-derived HCMV IL-10 protein is stabilized by covalent disulfide bond formation. However, no protein dimerization could be demonstrated under non-reducing immunoblot conditions (Figure 2B), indicating that disulfide bond formation does not occur in *E. coli* cells during protein translocation across the inner membrane and thereafter. Of note, it could be demonstrated that disulfide bridging of both monomers is not essential for dimerization [21], suggesting the possibility of a non-covalent assembly of both subunits as described for hIL-10. Thus, the lack of disulfide bond formation in the HCMV IL-10 recombinant protein does not necessarily exclude recovery of its biological activity. A covalent linkage of both monomers would, however, add to the stability of the protein, which would be highly desirable for a prospective *in vivo* application.

### Biological activity of *E. coli*-derived vIL-10 proteins

#### Recombinant vIL-10 proteins activate STAT3

The pivotal attribute of viral IL-10 proteins is their structural similarity to the host’s interleukin counterpart. As a result, vIL-10 analogues display potent immunosuppressive traits, a key factor of the immune escape mechanism of herpes viruses and the establishment of a latent infection [4]. Several downstream mechanisms after binding of vIL-10 to the host’s IL-10 receptor (IL-10R) as well as consequent immunomodulating effects have been described, which can be partly traced back to observable events at the level of cell signal cascade [22].

**Figure 3:**
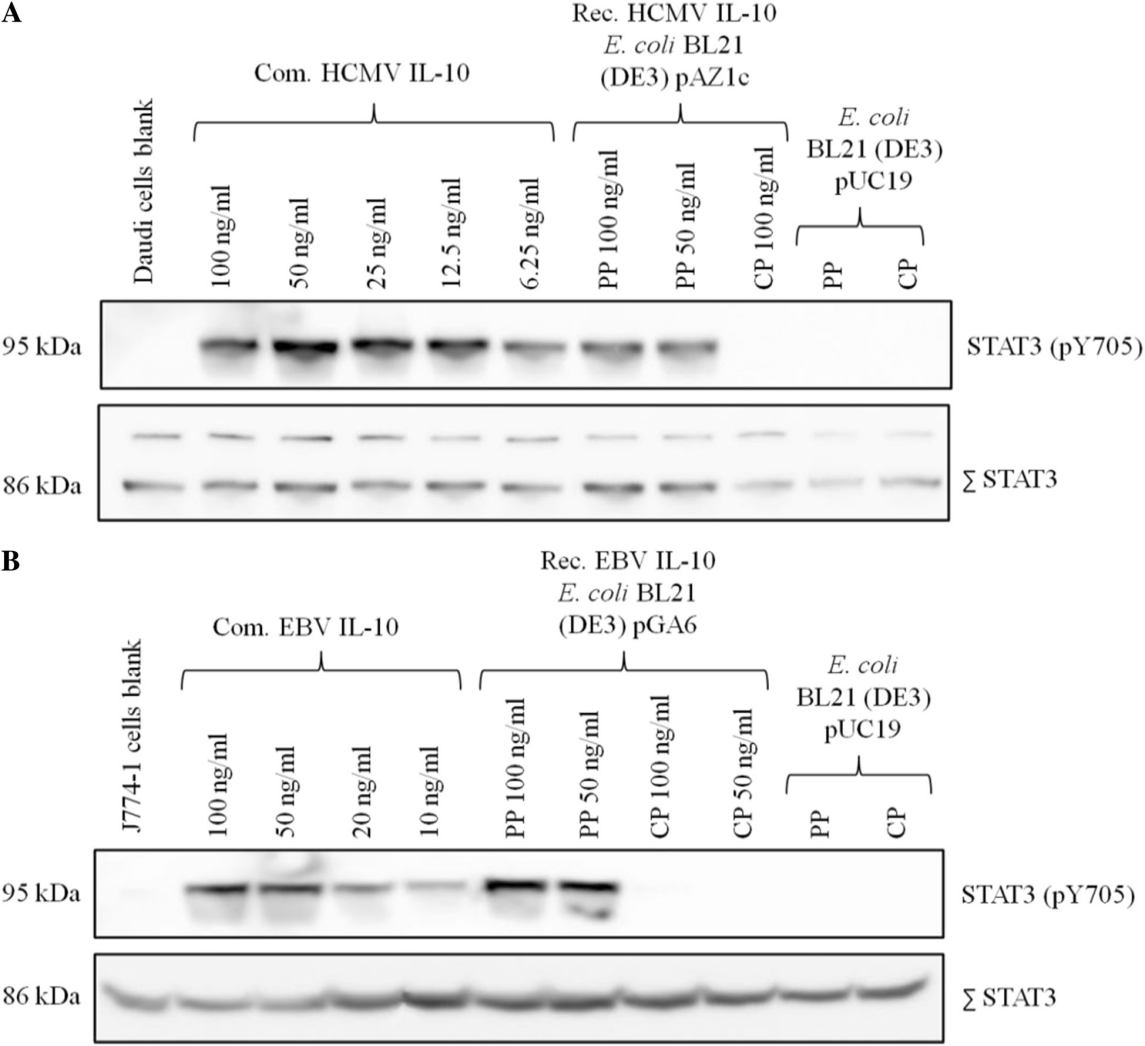
**Activation of STAT3 by *E. coli *derived viral IL-10.** STAT3 phosphorylation (STAT3-pY705) was analyzed by immunoblot of protein extracts from human Daudi cells (HCMV IL-10; **A)** and J774.1 mouse macrophages (EBV IL-10; **B)** treated with different concentrations of bacteria-derived viral IL-10. Total STAT3 was used to ensure equal protein loading in all lanes (double bands in Daudi cells represent STAT3 isoforms α and β). Periplasmic and cytoplasmic fractions of pUC19 transformed *E. coli* BL21 (DE3) cells and commercial viral IL-10 proteins served as controls. One experiment representative of two is shown. PP = periplasmic fraction; CP = cytoplasmic fraction; Com. = commercial; Rec. = recombinant.

One well established method to examine *in vitro* biological activity of IL-10, no matter of its origin, targets the recruitment of signal transducer and activator of transcription 3 (STAT3) as part of the signal transduction cascade after binding of IL-10 to its receptor [23]. Phosphorylation of STAT3 on aa Tyr_705_ indicates successful IL-10 – IL-10R interaction. We selected an *in vitro* cell assay using two different cell lines (murine macrophages J774.1 and Daudi’s Burkitt lymphoma cells) for testing the ability of *E. coli*-derived vIL-10 proteins to correctly induce the IL-10 signal transduction cascade. Since HCMV IL-10 is known to act only on cells of human origin due to high species specificity of HCMV IL-10 [24], we used Daudi’s Burkitt lymphoma cell line to assess the biological activity of recombinant HCMV IL-10. For recombinant EBV IL-10, the STAT3 assay was performed on a murine macrophage cell line since its function is not closely restricted to human species [25]. As positive controls, commercial recombinant EBV and HCMV IL-10 from R&D Systems were used. The commercially available viral IL-10 proteins showed phosphorylation of STAT3 after 1 h of incubation time and the intensity of immunoblot bands roughly reflected the IL-10 protein concentration loaded on the gel giving the assay not only a qualitative but also quantitative character (Figure 3). Periplasmic fractions of pAZ1c and pGA6 transformed *E. coli* BL21 (DE3) cells expressing either recombinant HCMV or EBV IL-10 were able to activate STAT3 to a level comparable to stimulation with the commercial vIL-10 proteins. This result indicates the *in vitro* biological activity of the *E. coli*-derived vIL-10 proteins secreted into the periplasm. It also implies a possible dimeric state of the secreted vIL-10 proteins which is compulsory for successful IL-10 – IL-10R interaction [26]. However, since an intermolecular disulfide bond formation could not be demonstrated, a possible non-covalent assembly of both HCMV IL-10 monomers must be suggested. The biological activity of recombinant vIL-10 proteins released into the culture supernatant could not be assessed due to low protein concentration.

Interestingly, the cytoplasmic fractions of pAZ1c and pGA6 transformed *E. coli* BL21 (DE3) cells showed no activation of STAT3 (Figure 3), thus being unable to induce the IL-10 signal transduction cascade. This observation confirms the presumption that most heterologous proteins expressed in the *E. coli* cytoplasm do not exist in a folded and thus functionally active form [27]. Intramolecular disulfide bridging is responsible for the high α-helical content of the vIL-10 protein structures, thus enabling dimer formation [13,20]. Since the cytoplasmic compartment lacks chaperones assisting in protein folding and does not provide a sufficient reducing milieu for disulfide bond formation, folding and dimerization of recombinant vIL-10 proteins is unlikely to occur in the bacterial cytoplasm [15]. As negative controls, culture supernatant, periplasmic and cytoplasmic fractions of pUC19 transformed *E. coli* BL21 (DE3) cells were used which did not trigger any phosphorylation of STAT3, demonstrating no other compound in the bacterial periplasm than the *E. coli*-derived vIL-10 proteins to be responsible for stimulation of IL-10R.

The results of the STAT3 *in vitro* cell assay indicate the correct folding and dimerization of recombinant vIL-10 proteins in the bacterial periplasm resulting in successful interaction with IL-10R and downstream STAT3 recruitment and activation.

#### Suppression of TNF-α secretion as proof of principle for biological activity of E. coli-derived EBV IL-10

**Figure 4:**
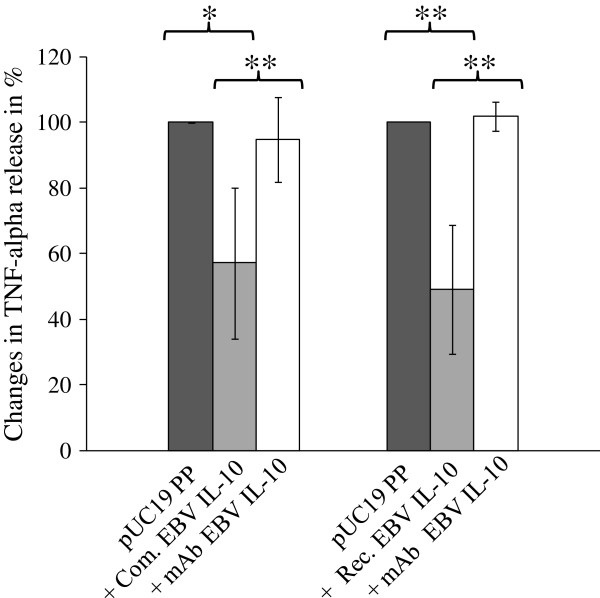
**Inhibition of LPS-induced TNF-α release by *E. coli *derived recombinant EBV IL-10.** J774.1 mouse macrophages were incubated with *E. coli* BL21 (DE3) pGA6 periplasmic fraction alone (bacterial recombinant EBV IL-10 at ~ 400 ng/ml) or in the presence of neutralizing monoclonal anti-EBV IL-10 antibody. Periplasmic fractions of *E. coli* BL21 (DE3) pUC19 were used as TNF-α induction control. Commercial EBV IL-10 (at ~ 400 ng/ml) served as positive control. TNF-α induction levels were set at 100%, and changes of TNF-α release are the means ± SD of four independent experiments. Statistical significance was determined using the Student t-test. Asterisks indicate statistically significant differences (* p ≤ 0.05; ** p ≤ 0.01) between pGA6 PP, pUC19 PP, and pGA6 after anti-EBV IL-10 treatment. PP = periplasmic fraction; Com. = commercial; Rec. = recombinant; mAb = monoclonal antibody.

One of the key characteristics of the IL-10 family is its anti-inflammatory properties leading to downregulation of proinflammmatory cytokine and chemokine expression [22]. Interaction of bacteria with adherent macrophages triggers the release of high amounts of the proinflammatory cytokine TNF-α [28]. Thus, reduced TNF-α secretion of lipopolysaccharide (LPS) stimulated macrophages can be used as proof of principle to confirm the functionality of recombinant IL-10 molecules produced by bacteria. Due to low secretory expression of recombinant HCMV IL-10 in *E. coli* BL21 (DE3), this read-out system was only tested with *E. coli*-derived EBV IL-10. Using the mouse macrophage cell line J774.1, TNF-α secretion was stimulated by sterile-filtered periplasm of pUC19 transformed *E.* coli BL21 (DE3) cells. Previous experiments with highly purified commercial LPS showed that absolute levels of TNF-α in the supernatant were too low to properly assess a significant reduction after IL-10 addition. However, the LPS content of the periplasmic fraction of pUC19 transformed *E. coli* BL21 (DE3) cells grown overnight to similar OD_600_ values proved to be stable enough for the induction of consistent TNF-α levels at 560.3 ± 315.0 pg/ml in the culture supernatant of J774.1 cells (data not shown). Both, the periplasmic fraction of pGA6 transformed *E. coli* BL21 (DE3) cells containing the secreted from of recombinant EBV IL-10 as well as the commercial analogue (R&D Systems) were able to significantly suppress TNF-α secretion of LPS-stimulated J774.1 mouse macrophages after 4 h of incubation (Figure 4). Compared to the TNF-α concentrations in the culture medium of the induction controls, periplasmic fractions of pGA6 transformed *E. coli* BL21 (DE3) cells were able to reduce the TNF-α level by 49 ± 20%. These values were within the same range of the commercial EBV IL-10 standard, which suppressed TNF-α levels by 57 ± 23%. Pre-incubation of either the periplasmic fraction of pGA6 transformed *E. coli* BL21 (DE3) cells or the commercial EBV IL-10 with an EBV IL-10 specific neutralizing antibody (R&D Systems) abrogated the suppression of TNF-α secretion, thus indicating an exclusive dependence of reduction of TNF-α release on the secreted interleukin. The results of the TNF-α cell assay further substantiate our hypothesis that recombinant EBV IL-10 transported into the periplasmic space of *E. coli* BL21 (DE3) cells shows *in vitro* biological activity.

## Conclusions

The results presented in this study demonstrate that *E. coli* cells are a suitable chassis for secretory expression of *in vitro* biologically active vIL-10 analogues. Due to the potent anti-inflammatory traits of these molecules, *E. coli* cells genetically modified for secretory expression of cytokines may be used as carriers for *in situ* intestinal cytokine delivery. The study clearly demonstrates that recovery of recombinant vIL-10 proteins with biological activity is only possible when the proteins were trapped in the bacterial periplasm. This phenomenon was also observed for the expression of hIL-10 in a previous study [10]. Furthermore, our results demonstrate that the Sec-dependent *E. coli* OmpF leader peptide is a suitable export signal for other members of the IL-10 subfamily although the viral IL-10 proteins only share 27 – 83% amino acid sequence identity with hIL-10 [13,19]. Other attempts using bacterial transporters, which enable a direct secretion of recombinant IL-10 into the culture medium such as the *E. coli* hemolysin system, did not result in successful secretion of a biologically active molecule [29]. Consequently, the best strategy in the development of a carrier for the *in situ* intestinal IL-10 delivery seems to be the periplasmic production of the protein and its subsequent release from the carrier via a cell lysis system. For future *in vivo* applications, a controlled release of the recombinant vIL-10 proteins as well as the biological containment of the genetically modified bacteria is mandatory. For example, Kong et al. have developed an arabinose-dependent lysis system for the *in vivo* delivery of vaccine antigens [11] which combines both release of bacteria expressed proteins and containment of genetically engineered bacterial carriers. The realization of such a construct for IL-10 would then blaze the trail for testing IL-10 expression in a murine IBD model. In this way, IBD might be prospectively treated in a selective and strict topical manner, thus avoiding systemic side effects as known from other immunosuppressive drugs.

## Methods

Restriction enzymes, proof-reading Phusion DNA polymerase and T4 DNA ligase were purchased from New England Biolabs (Beverly, MA, USA) and were used according to the manufacturer’s instructions. GoTaq DNA polymerase was from Promega (Madison, WI, USA). DNA sequencing kits (ABI PRISM BigDye Terminator v3.1 Cycle Sequencing Kit) were obtained from Applied Biosystems (Darmstadt, Germany). Oligonucleotide primers were synthesized by biomers.net (Ulm, Germany). Kits for plasmid mini-prep and PCR purification as well as DyeEx 2.0 Spin Kits for clean-up of sequencing reaction were all purchased from QIAGEN (Hilden, Germany). DNA gel extraction kits were from Omni Life Science (Bremen, Germany). Prestained molecular mass marker BenchMark® for SDS-PAGE electrophoresis and nitrocellulose membranes for protein blotting were obtained from Life Technologies (Darmstadt, Germany). Reducing sample buffer containing 100 mM dithiotreitol as well as a detection kit for enhanced chemiluminescence (ECL SuperSignal West Pico Substrate) was procured by Thermo Fisher Scientific (Dreieich, Germany). Vivaspin 20 concentrators (10000 MWCO PES) for enrichment of recombinant vIL-10 proteins in different cell fractions were purchased from Sartorius (Göttingen, Germany). Culture medium components were supplied from BD (Franklin Lakes, NJ, USA). J774.1 cells were cultivated in complete Iscove’s medium procured from Biochrom (Berlin, Germany) supplemented with 10% (v/v) FCS while DAUDI cells were grown in RPMI 1640 liquid medium with stable glutamine containing 20% (v/v) FCS. All cell lines were maintained at 37°C in 5% CO_2_/95% air. Other chemicals obtained from commercial sources were all of analytical grade and were used without further purification.

### Bacterial strains, plasmids and growth conditions

Cloning experiments were performed in *Escherichia coli* MDS 42 [30] obtained from Scarab Genomics (Madison, WI, USA) while protein expression analysis was carried out in *Escherichia coli* BL21 (DE3) [F^-^*ompT hsdS*_*B*_ (r_b_ ^-^ m_B_ ^-^) *gal dcm* (λDE3)] obtained from New England Biolabs. Plasmids used in this study were pUC19 (New England Biolabs,), pGA4 and pGA6 (GeneArt). Cells were generally grown aerobically either in liquid (shaking) or on solid Luria-Bertani (LB) medium (1% bactotryptone, 0.5% yeast extract, and 0.5% sodium chloride) [31] at 37°C. Media were supplemented when appropriate with ampicillin (100 μg/ml; Sigma Aldrich, St. Louis, MO, USA).

### DNA sequencing

Nucleotide sequencing of the modified transporter construct inserted in pAZ1c and pAZ1e expression plasmids was performed following the application of automated DNA sequencing method based on the dideoxy chain termination method [32], using M13 primers for both plasmids.

### Transformation of *E. coli* strains

*E. coli* strains were made competent and transformed using standard techniques [33]. Briefly, either a 20-ng aliquot of the ligation mixture or the corresponding sequenced plasmid was added to 40 μl of electrocompetent *E. coli* cells, and the mixed solution was transferred into an ice-cold 0.2-cm disposable cuvette (Bio-Rad Laboratories, Hercules, CA, USA). Electroporation was conducted using a Gene Pulser Xcell electroporation system (Bio-Rad Laboratories) at 2.5 kV, 25 μF, and 400 Ω and was immediately followed by the addition of 1 ml of SOC medium (SOB medium containing 20 mM glucose, 10 mM MgSO_4_, and 10 mM MgCl_2_). The culture was then incubated for 1 h at 37°C. 10 μl and 100 μl aliquots as well as the rest volume were spread onto LB agar containing 100 μg/ml ampicillin and incubated overnight at 37°C to select for the antibiotic-resistant recombinants. Successful transformants were confirmed by mini-prep growth with plasmid extraction followed by restriction digestion and gel electrophoresis.

### Construction of the EBV and HCMV IL-10 expression vectors

Molecular methods were performed as described by Sambrook et al. [34]. To construct plasmids pGA4 and pGA6, 700 and 664 bp DNA fragments including the T7 promoter and the first 66 nucleotides of the *E. coli ompF* gene (NC_000913.2) encoding the signal peptide sequence fused in frame to *E. coli* codon optimized genes of the mature form of either HCMV IL-10 (1LQS_M) or EBV IL-10 (YP_401634) were de novo synthesized and cloned into *Sac*I/*Kpn*I digested pUC-derived vector pMA by GeneArt. The pGA4 and pGA6 plasmids were *Eco*RI digested and the resulting DNA fragments including the vIL-10 transporter construct and the T7 promoter region were subsequently ligated into *Eco*RI digested pUC19 resulting in the expression plasmids pAZ1c and pAZ1e.

### Viral IL-10 sample preparation

*E. coli* BL21 (DE3) cells transformed with the HCMV and EBV IL-10 expression plasmids pGA4, pGA6, pAZ1c and pAZ1e were grown overnight at 37°C in 50 ml of LB broth containing 100 μg/ml of ampicillin. From overnight cultures, the culture supernatant was removed and sterile filtered (0,2 μm, Filtropur S; Sarstedt, Nümbrecht, Germany). The periplasmic fraction was prepared according to the method of Neu et al. [12]. Briefly, the cell pellet was resuspended in ice-cold protoplast buffer containing 30 mM Tris–HCl, 40% sucrose (w/v), and 2 mM EDTA at 1/10 of the culture volume. After 15 min of incubation on ice, the cells were centrifuged at 5000 rpm for 60 min at 4°C. The supernatant fluid was discarded, and the pellet was resuspended in a volume of cold water equal to that of the original volume of the suspension. After 15 min of incubation on ice and centrifugation, the supernatant fluid equivalent to the periplasmic fraction was collected. The remaining pellet resembling the cells’ cytoplasm was washed twice, resuspended in cold water and subsequently centrifuged. To solubilize the cytoplasmic fraction, the washed pellet was resuspended in cold water again, treated constantly for two minutes by sonification using Sonopuls HD 70 (Bandelin electronic, Berlin, Germany) and subsequently centrifuged. The procedure was repeated until the visible pellet was gauzy.

### Viral IL-10 sandwich ELISA

Recombinant HCMV IL-10 levels were quantified by using an in-house ELISA according to Chang’s description [14]. Briefly, 96 well microplates (#2592; Corning Life Sciences, Corning, USA) were coated with anti-HCMV IL-10 polyclonal antibodies (R&D Systems) at a concentration of 1 μg/ml overnight at 4°C. After washing three times with PBS containing 0.05% Tween 20 (AppliChem, Darmstadt, Germany), plates were blocked with 3% (w/v) bovine serum albumin in PBS at 37°C for 1 h followed by three washing steps. Bacterial samples as well as commercial recombinant HCMV IL-10 (R&D Systems) used as standard at a concentration range from 5 ng/ml to 312.5 pg/ml were incubated for 1 h at 37°C. After washing four times, wells were incubated with 1 μg/ml anti-HCMV IL-10 biotinylated antibodies (R&D Systems) for 1 h at room temperature. After four washing steps, Streptavidin horseradish peroxidase (HRP) conjugate (R&D Systems) at a dilution of 1:2000 was incubated at room temperature for 30 minutes followed by six washing steps. Finally, tetramethylbenzidine (TMB) substrate (Life Technologies) was added at room temperature, and the colorimetric reaction was stopped after 30 min with 1 N hydrochloric acid. Intra-assay and inter-assay variance were calculated 4.4% and 5.5%, respectively.

The concentration of recombinant EBV IL-10 was measured using the Quantikine® kit for human IL-10 from R&D Systems. The assay was carried out according to the manufacturer’s instructions.

All samples were measured in duplicate at 450 nm with wavelangth correction of 540 nm. Cytokine levels were determined by linear regression analysis using a standard curve generated with the supplied calibration material in case of EBV IL-10 or the commercial recombinant HCMV IL-10 protein standard (R&D Systems), respectively. As negative controls served supernatant and periplasmic fraction from pUC19 transformed *E. coli* BL21 (DE3). The vIL-10 concentrations are the result of three independent experiments and presented as mean ± SD.

### SDS PAGE vIL-10 immunoblot analysis

Enrichment of recombinant vIL-10 from periplasmic fractions of pAZ1c and pGA6 transformed *E. coli* BL21 (DE3) cells was performed with Vivaspin 20 concentrators (exclusion limit 10000 MWCO) obtained from Satorius Stedim Biotech (Göttingen, Germany). Periplasmic fractions were separated by SDS-PAGE on a 10% (w/v) NuPAGE Novex Bis-Tris gel (Life Technologies) prior to blotting onto nitrocellulose which was blocked for 1 h with blotting solution consisting of 25 mM Tris, 0.15 M NaCl and 0.1% Tween 20 at pH 7.4 supplemented with 3% (w/v) BSA. Membranes were incubated overnight at 4°C with either a goat anti-HCMV IL-10 biotinylated antibody (R&D Systems; 100 ng/ml diluted at 1:500) to detect HCMV IL-10 or a mouse anti-EBV IL-10 antibody (R&D, Minneapolis, USA; 2 μg/ml diluted at 1:250) to detect EBV IL-10. After washing, a second 1 h incubation step followed at room temperature with either a HRP-conjugated goat anti-mouse immunoglobulin G (IgG) (R&D, Minneapolis, USA) diluted at 1:1000 for detection of EBV IL-10 or with streptavidin HRP conjugate (R&D, Minneapolis, USA) at a dilution of 1:2000 for detection of HCMV IL-10.

Chemoluminescence was detected by incubation with TMB-H_2_O_2_ as substrate (Super Signal West Pico Chemoluminescent Substrate Kit, Thermo Scientific) according to the manufacturer’s manual and subsequent analysis with a LAS 3000 (FUJIFILM Europe, Düsseldorf, Germany) bioimager. Commercial vIL-10s (R&D Systems) were used as standard. SDS-PAGE immunoblot data were the result of at least two independent experiments.

### *In vitro* assays for characterization of vIL-10 biological activity

Biological activity of recombinant EBV IL-10 was tested on murine macrophage cell line J774.1, whereas for recombinant HCMV IL-10 the human Burkitt lymphoma DAUDI cell line were used.

a) *STAT3 cell assay*

Cells were incubated for 30 min at 5 × 10^6^ per well with recombinant vIL-10 from the periplasmic fraction of either pAZ1c or pGA6 transformed *E. coli* BL21 (DE3) cells, then harvested and treated with cell lysis buffer (RIPA buffer; Sigma Aldrich) containing inhibitors of proteases (Complete Mini; Roche Applied Science, Mannheim, Germany) and phosphatases (Phosphostop; Roche Applied Science, Mannheim, Germany) for 1 h on ice. Cell lysates were clarified by centrifugation, and the supernatants were then analyzed by SDS-PAGE on a 10% (w/v) NuPAGE Novex Bis-Tris gel (Life Technologies) and subsequent Western blotting with polyclonal antibodies directed against either phospho-STAT3 (Y705) (Cell Signaling Technology, Danvers, MA, USA) or total STAT3 (C-20; Santa Cruz Biotechnology, Santa Cruz, CA, USA), both diluted at 1:1000 in TBS buffer supplemented with 0.1% (v/v) Tween 20 and 5% (w/v) BSA. HRP-conjugated goat anti-rabbit IgG (Jackson ImmunoResearch, West Grove, PA, USA) diluted at 1:15000 in TBS-T buffer containing 3% BSA was utilized for detection. Mild stripping was performed using a buffer containing 1.5% glycin (w/v), 0.1% sodium dodecylsulfate and 1% (v/v) Tween 20 in aqua dest. at pH 2.2 – 2.5. The periplasmic fraction of pUC19 transformed *E. coli* BL21 (DE3) and commercial vIL-10 (R&D Systems) were used as controls. STAT3 immunoblot data were the result of at least two independent experiments.

b) *TNF-α cell assay for E. coli-derived EBV IL-10*

Prior to the experiment, J774.1 cells were seeded at a density of 5 × 10^4^/ml in tissue culture plates and were then incubated for 2 h at 37°C. Recombinant EBV IL-10 from the periplasmic fraction of pGA6 transformed *E. coli* BL21 (DE3) cells was either preincubated alone or in the presence of 3 μg/ml neutralizing anti-EBV IL-10 monoclonal antibody (R&D Systems) for 20 min at 37°C, filled up to equal volumes with LPS containing periplasmic fraction of pUC19 transformed *E. coli* BL21 (DE3) cells, and then added to J774.1 cells at a concentration of ~ 400 ng/ml. After 4 h of incubation at 37°C, the amount of TNF-α released in the medium was measured in duplicate by ELISA (R&D Systems) according to the manufacturer’s instructions. Periplasmic fraction of pUC19 transformed *E. coli* BL21 (DE3) was used as TNF-α induction control. Commercial EBV IL-10 (R&D Systems) served as positive control. Presented values were the result of four independent experiments.

## Abbreviations

aa: Amino acid; EBV: Epstein-Barr virus; ELISA: Enzyme linked immunosorbent assay; HCMV: Human cytomegalovirus; hIL-10: Human interleukin-10; HRP: Horseradish peroxidase; IBD: Inflammatory bowel disease; IL-10: Interleukin-10; IL-10R: Interleukin-10 receptor; LPS: Lipopolysaccharide; OD: Optical density; OmpA: Outer membrane protein A; OmpC: Outer membrane protein C; OmpF: Outer membrane protein F; PCR: Polymerase chain reaction; PelB: Pectate lyase B; PhoA: Alkaline phosphatase; STAT3: Signal transducer and activator of transcription 3; StII: Heat-stable enterotoxin 2; TMB: Tetramethylbenzidine; TNF-α: Tumor necrosis factor alpha; Tyr: Tyrosine; vIL-10: Viral interleukin-10.

## Competing interests

The authors declare that they have no competing interests.

## Authors’ contributions

CP, DSM, AZ, FRB, KZ, and FG conceived of the study and CP, DSM, AZ, and FG designed the experiments. SF performed the experiments, MB finalized the experimental data. SF, MB, CP, and FG analyzed and interpreted the data. SF, CP, and FG wrote the paper. All authors read and approved the final manuscript.
